# Specimen retrieval approaches in patients undergoing laparoscopic colorectal resections: a literature-based review of published studies

**DOI:** 10.1093/gastro/gou053

**Published:** 2014-08-21

**Authors:** Muhammad S. Sajid, Muhammad I. Bhatti, Parv Sains, Mirza K. Baig

**Affiliations:** ^1^Department of General & Laparoscopic Colorectal Surgery, Worthing Hospital, Worthing, UK and ^2^Department of General Surgery, Queen Elizabeth Hospital, Kings Lynn NHS Foundation Trust, Kings Lynn, UK

**Keywords:** colorectal cancer, laparoscopic colorectal surgery, umbilical incision, transverse incision, anal retrieval, vaginal retrieval

## Abstract

**Objective:** To review the published studies reporting various specimen retrieval incisions being used by colorectal surgeons in patients undergoing laparoscopic colorectal resections (LCR).

**Methods:** Standard medical electronic databases were searched to find relevant articles and a summary conclusion was generated.

**Results:** There were 43 studies reporting various approaches used for the purpose of specimen retrieval in 2388 patients undergoing LCR. The most common approaches were periumbilical midline incision (1260 reported case in the literature), transverse incision (583 reported cases in the literature) in the right- or left iliac fossa, depending on the side of colonic resection, and Pfannensteil incision (293 reported cases in the literature). Periumbilical midline incision was associated with the higher risk of developing incisional hernia (odds ratio 53.72; 95% confidence interval 7.48–386.04; *Z* = 3.96; *P = *0.0001). In terms of surgical site infection (SSI), there was no difference between the three common approaches to specimen retrieval. Transanal and transvaginal approaches were associated with higher risk of SSI.

**Conclusions:** Midline, transverse and Pfannensteil incisions were the most commonly used approaches for specimen retrieval following LCR. Midline incision was associated with higher risk of incisional hernia. Risk of SSI was similar in all three common approaches. The transanal and transvaginal approaches pose a higher risk of SSI. These conclusions are based on the combined outcome of published case series, case reports and comparative studies. Randomized, controlled trials with longer follow-up are required before recommending the routine use of any approach for specimen retrieval in patients undergoing LCR.

## INTRODUCTION

Various types of incisions used in abdominal operations are an important source of post-operative morbidities such as pain, surgical site infections, scarring, tumour implantation and incisional hernia [1]. One of the objectives of laparoscopic- or other minimally invasive surgical approaches is to minimise incision-related complications and to improve post-operative outcomes. Laparoscopic colorectal surgery, as compared with open surgery, has been reported to improve short-term and long-term outcomes in patients suffering from various colorectal disorders. Laparoscopic colorectal resection (LCR) has therefore become a preferred technique for treating both benign and malignant conditions of the colon and rectum [2–7]. After a successful dissection in laparoscopic colorectal surgery, the enlargement of a trocar incision, resulting in ‘minilaparotomy', is invariably necessary for two major reasons; firstly for intestinal anastomosis, to maintain the continuity of the gastro-intestinal tract, and secondly for the purpose of retrieval of the specimen. The extension of a port-site incision causes more tissue trauma than one would expect from a smaller port wound and thus potentially reduces the aforementioned advantages of LCR [8]. This poses a special challenge to operating surgeons, due to the size of the specimen and the desire to keep the retrieval incision as small as possible to retain the benefits of laparoscopic surgery. In addition, the potential problems of dissemination of tumour cells, implantation of tumour cells in the wound; metastasis and wound contamination must be kept in mind during the process of specimen retrieval [9]. A number of solutions have been reported, for the purpose of avoiding minilaparotomy altogether or placing another incision away from the port incisions to retrieve the specimen. These include transverse incision in the left iliac fossa, transverse incision in the right iliac fossa, McBurney’s incision, extension of the umbilical port incision in midline, and stoma site incision. Additionally, specimen retrieval through natural orifices—such as through the anus or vagina—has also been reported as a relatively preferable solution.

The objective of this article is to review the various specimen retrieval techniques reported in the medical literature during LCR and generate a summary conclusion based on the level of evidence available.

## METHODS

### Data sources

All the published articles on specimen retrieval techniques during laparoscopic colorectal resection were identified through searches of the Medline, Embase, CINAHL, Cochrane library and Pubmed databases. The search terms “colorectal surgery”, “laparoscopic”, “minimal invasive surgery”, “natural orifice retrieval”, “trocar incision”, “midline incision”, “periumbilical incision”, “Pfannensteil incision” and “transverse incision” were used alone and in various combinations. Relevant articles referenced in these publications were also downloaded from databases. The ‘related article' function was used to widen the search results. All abstracts, case reports, case series and published single-centre or multi-centre studies were retrieved and searched comprehensively.

### Study selection

For inclusion in the literature review, a study had to meet the following criteria: (i) randomized, controlled trial, case controlled trial, cohort studies, all types of comparative studies, case series and case reports, (ii) laparoscopic colorectal resections for both benign and malignant conditions, (iii) evaluation of surgical site infection rate, and (iv) trials in patients undergoing any kind of surgery.

### Data extraction

Using a predefined data format, two independent reviewers (MSS and MIB) extracted data from each study, which resulted in high and satisfactory interobserver agreement. Information collected included the name(s) of the author(s), title of the study, journal in which the study was published, country and year of the study, treatment regimen, length of the therapy, method by which specimens were retrieved, testing sample size (with sex differentiation if applicable) and the number of patients receiving each regimen. Within each arm in case of comparative study, the reviewers noted the number of patients who responded to- and the number of patients who failed to respond to treatment, the patient compliance rate in each group, the number of patients reporting complications and the number of patients with absence of complications. After completing the data extraction, the two independent reviewers discussed the results and, if discrepancies were present, a consensus was reached.

### Data synthesis and statistical analysis

Where applicable, the RevMan 5.2 software package [10, 11], provided by the Cochrane Collaboration, was used for the statistical analysis to achieve a combined outcome. The odds ratio (OR) with a 95% confidence interval (CI) was calculated for binary data. The random- and fixed-effects models (where applicable) were used to calculate the combined outcomes of both binary and continuous variables [12, 13]. If the standard deviation was not available, then it was calculated according to the Cochrane Collaboration's guidelines [10]. This process involved assumptions that both groups had the same variance—which may not have been true—and variance was either estimated from the range or from the *P*-value. The estimate of the difference between the two techniques was pooled, depending upon the effect weights in results determined by each trial estimate variance. A forest plot was used for the graphical display of the results. The square around the estimate stood for the accuracy of the estimation (sample size), and the horizontal line represented the 95% CI.

## RESULTS

**Figure 1.**
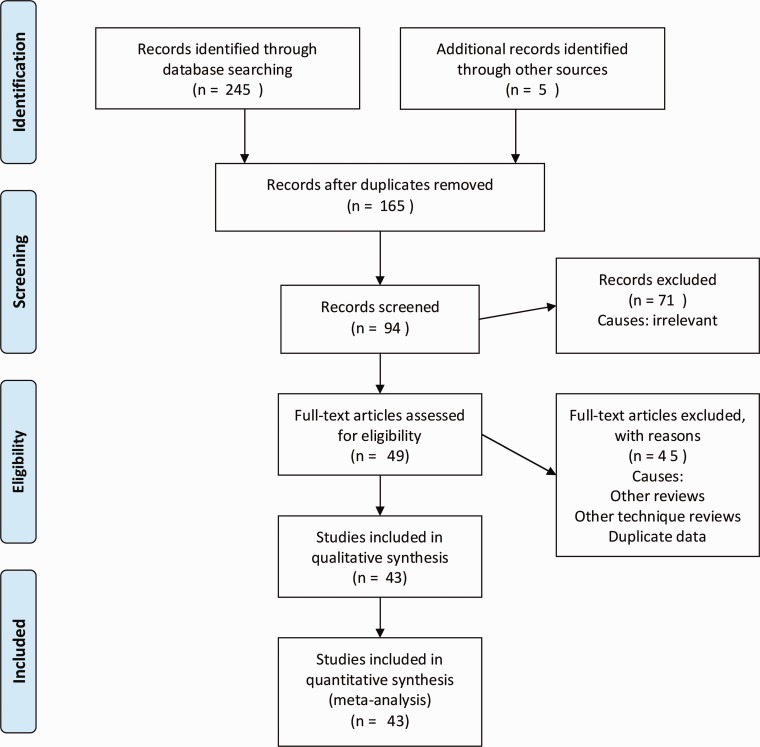
PRISMA flow diagram.

There were 43 studies reporting various approaches used for the purpose of specimen retrieval in 2388 patients undergoing LCR [14–56]. These approaches can be categorized as transvaginal, transanal, transverse incision in the right or left iliac fossa, periumbilical midline incision, Pfannensteil incision and approach through the stoma site. The literature search strategy and methodology is given in Figure 1.

### Studies reporting single technique

#### 1) Transvaginal approach

Thirteen studies [14–26] on 143 patients reported on the use of transvaginal approach to retrieve the specimen after LCR. These included five case reports [16–18, 23, 26] and eight case series [14, 15, 19–22, 24, 25]. There was no reported incision site herniation or tumour recurrence among these 143 patients. Overall, there were eight patients (5.5%) with various complications including surgical site infection (SSI) (Table 2).

**Table 1.** Characteristics and variables of articles reporting transvaginal specimen retrieval in patients undergoing laparoscopic colorectal surgery

Trials	Year	Country	Study type	Patient number	Age (years)	Surgery details	Follow up (months)	Incisional hernia	Complications/ incision site infection	Recurrence	Use of wound protector
Awad *et al.* [14]	2011	USA	Case series	14	62 (range 50–80)	Right hemicolectomy for both benign and malignant conditions	17.8 (range 8–32)	0	0	0	Hubert bag
Boni *et al.* [15]	2007	Italy	Case series	11	45 ± 12	Rectal resection for benign conditions	4 ± 2	0	0	0	Standard vaginal extractor with endobag
Dozois et [16]	2008	USA	Case report	1	53	Hysterectomy	1	0	0	0	No
						Salpingo-ophorectomy					
						Total colectomy					
Franklin *et al.* [17]	2008	USA	Case report	1	88	Right hemicolectomy for caecal carcinoma	1 week	0	0	0	Specimen bag
García–Flórez *et al.* [18]	2010	Spain	Case report	1	86	Anterior resection with en bloc salpingo-ophorectomy for sigmoid carcinoma	3	0	0	0	Plastic retractor
Ghezzi *et al.* [19]	2007	Italy	Case series	33	33.4 (range 25–43)	Rectosigmoid resection for endometriosis	13 (range 3–27)	0	1 case of pelvic seroma	0	Retrieval bag
McKenzie *et al.* [20]	2010	USA	Case series	4	74 (range 68–81)	Right hemicolectomy for both benign and malignant conditions	3	1	1 case of internal hernia not related to incision	0	Specimen bag
Palanivelu *et al.* [21]	2008	India	Case series	7	49.5 (range 34–65)	Proctocolectomy for colorectal cancer and familial adenomatosis coli	12	0	1 case of pouchitis	0	Endobag
									1 case of anastomotic leak		
Park *et al.* [22]	2010	South Korea	Case series	14	66 (range 44–74)	Colectomy for colorectal carcinoma	34	0	0	1 case of distant metastasis	Plastic bag
Sanchez *et al.* [23]	2009	USA	Case report	1	63	Sigmoid colectomy for rectal prolapse	3	0	0	0	No
Tarantino *et al.* [24]	2011	Switzerland	Case series	34	64 (range 35–88)	Anterior resections for diverticular disease	6	0	1 case of wound dehiscence	0	Ring wound protector
									1 case of colpitis		
									1 case of anastomotic leak		
									2 cases of wound haematoma		
Torres *et al.* [25]	2012	Argentina	Case series	21	50 (range 32–69)	Colectomy for both benign and malignant conditions	34	0	0	0	Alexis wound retractor
Wilson *et al.* [26]	2007	UK	Case report	1	84	Right hemicolectomy for hepatic flexure carcinoma	1	0	0	0	Hubert bag

**Table 2.** Characteristics and variables of articles reporting transanal specimen retrieval in patients undergoing laparoscopic colorectal surgery

Trials	Year	Country	Study type	Patient number	Age (years)	Surgery details	Follow up (months)	Incisional hernia	Complications/incision site infection	Recurrence	Use of wound protector
Akamatsu *et al.* [27]	2009	Japan	Case series	16	n/a	Anterior resection for sigmoid carcinoma	2–15	0	0	0	No
Awad *et al.* [28]	2012	USA	Case report	1	27	Colonic resections for benign disorders	1	0	0	0	No
Cheung *et al.* [29]	2009	China	Case series	10	66 (range 55–81)	Left colonic resections for carcinoma	1	0	0	0	TEO device
Co *et al.* [30]	2010	China	Case report	1	80	Left colonic resection for carcinoma	1	0	0	0	TEO device
Franklin *et al.* [31]	2012	USA	Case series	179	66.9 ± 14.4	Anterior resection for rectal cancer	24	0	3 cases of anastomotic leakage	9	Plastic bag
									3 cases of anal stenoses		
Fuchs *et al.* [32]	2012	Germany	Case series	14	61 (range 28–86)	Colonic resection for benign conditions	6	0	0	0	TEO device
Hara *et al.* [33]	2011	Japan	Case series	8	71 (range 48–75)	Anterior resection for rectal carcinoma	1	0	0	0	No
Knol *et al.* [34]	2009	Belgium	Case report	1	20	Rectal resection for benign condition	1	0	0	0	Novymed proctoscope
Lacy *et al.* [35]	2012	Spain	Case report	1	36	Colectomy for ulcerative colitis	1	0	0	0	Endo Catch II
Leroy *et al.* [36]	2011	France	Case series	16	61.2	Anterior resection for diverticular disease	1	0	0	0	No
Makris *et al.* [37]	2012	USA	Case report	1	n/a	n/a	n/a	n/a	n/a	n/a	No
Nishimura *et al.* [38]	2011	Japan	Case series	18	46–84	Anterior resection for colorectal cancer	5–20	0	1 case of anastomotic leakage	0	Alexis wound retractor
									1 case of umbilical port infection		
Ooi *et al.* [39]	2009	Singapore	Case report	1	51	Anterior resection for rectal cancer	1	0	0	0	No
Saad *et al.* [40]	2011	Germany	Case series	15	61 (range 46–76)	Anterior resection for both benign and neoplastic conditions	1	0	0	0	TEO device
Saad *et al.* [41]	2010	Germany	Case series	8	n/a	Anterior resection for both benign and neoplastic conditions	1	0	0	0	McCartney tube
Wolthius *et al.* [42]	2011	Belgium	Case series	21	41 (34–66)	Anterior resection for both benign and neoplastic conditions	3.6	0	1 case of anastomotic leakage	0	Specimen retrieval pouch

n/a = not available, TEO = transanal endoscopic operation.

#### 2) Transanal approach

A transanal approach to retrieve the specimen after LCR was reported in sixteen case series [27–42] recruiting 311 patients. Nine patients (2.9%) developed various complications, set out in Table 2. The risk of developing post-operative complications was higher following a transvaginal approach than with a transanal approach (OR 0.50; 95% CI 0.19–0.33).

#### 3) Transverse incision through the right or left iliac fossa

Jones *et al.* [43] published data on 500 patients undergoing LCR for diverticular disease, where the specimen was retrieved through a transverse incision in the left iliac fossa (Table 3). Risk of incisional hernia was 0.4%; that for SSI was 1.2% and risk of reported anastomotic leak was 1.4%.

**Table 3.** Characteristics of studies reporting various other approaches of specimen retrieval in patients undergoing laparoscopic colorectal surgery

Trials	Year	Country	Study type	Approach of specimen retrieval	Patient number	Age (years)	Follow up (months)	Surgery details	Incisional hernia	Complications/ incision site infection	Recurrence	Use of wound protector
Jones *et al.* [43]	2008	Australia	Case series	Transverse incision in the left iliac fossa	500	58	n/a	All types of colonic resection for diverticular disease	2	7 cases of anastomotic leakage	0	No
										6 cases of wound infections		
Casciola *et al.* [44]	2008	Italy	Case series	Periumbilical midline incision	352	n/a	6	69 splenectomies	1	3	n/a	Endobag
								138 right hemicolectomies				
								115 gastric resections				
López-Köstner *et al.* [45]	2008	Chile	Case series	Periumbilical midline incision	106	54	27	Sigmoid colectomy for diverticular disease	3	3	1	No
Wilhelm *et al.* [46]	2006	Germany	Case series	Pfannensteil incision	100	58	19	Sigmoid colectomy for diverticular disease	1	11	1 diverticulitis	n/a
Sahakitrungruang *et al.* [47]	2008	Thailand	Case series	Pfannensteil incision	7	na	1	Proctocolectomy for ulcerative colitis	0	0	0	n/a

n/a = not available.

#### 4) Periumbilical midline incision

Two studies [44, 45] reported data on 458 patients undergoing colorectal and upper gastro-intestinal surgical resections where the specimen was retrieved through a periumbilical (extended port side wound) midline incision (Table 3). There were four cases of incisional hernia (0.87%), six cases of SSI (1.3%) and one case of distant recurrence. The risk of developing incisional hernia was greater in cases of periumbilical midline incision than with left iliac fossa transverse incision (OR 2.19; 95% CI 0.40–12.3).

#### 5) Pfannensteil incision

Two articles reported on the use of Pfannensteil incision for specimen retrieval in 100 patients undergoing laparoscopic sigmoid colectomy for diverticular disease [46], and in seven patients undergoing laparoscopic panproctocolectomy for ulcerative colitis [47] (Table 3). There was only one case of incisional hernia (0.93%) and 11 cases of SSI in this case series [46].

### Studies reporting comparison between two techniques

#### 1) Transanal vs. periumbilical midline incision approach

This approach was published in two comparative studies [48, 49], in which 68 patients underwent LCR for benign colorectal conditions (Table 4). Statistically, the duration of operation [Standardized Mean Difference (SMD) 1.81; 95% CI -1.99–5.62; *Z = *0.93; *P = *0.35] and risk of incisional hernia (OR 6.46; 95% CI 0.24–174.08; *Z = *1.11; *P = *0.27) were similar (SMD 1.81; 95% CI -1.99–5.62; *Z = *0.93; *P = *0.35) in both approaches. However, the transanal approach was associated with higher risk of SSI (OR 17.40; 95% CI 1.50–202.47; *Z = *2.28; *P = *0.02) (Figure 1).

**Table 4.** Characteristics and variables of studies reporting comparisions between various approaches of specimen retrieval in patients undergoing laparoscopic colorectal surgery

Trials	Year	Country	Study type	Approach of specimen retrieval	Patient number	Age (years)	Surgery details	Operation time (minutes)	Follow-up (Months)	Hernia	Infection/ complications	Recurrence
Christoforidis *et al.* [48]	2012	Switzerland	Case control	Transanal	10	47 (range 26–62)	Left colonic resections for benign disease	200 ± 60	n/a	1	0	0
				Periubmilical midline incision	20	56 (range 38–81)		205.5 ± 49		0	0	0
Eshuis *et al.* [49]	2010	Netherlands	Case control	Transanal	8	31 (range 19–61)	Ileocolic resections for inflammatory bowel disease	208 ± 45.1	3	0	3	0
				Periubmilical midline incision	30			115 ± 15.1		0	1	0
Lee *et al.* [50]	2012	Canada	Case control	Periumbilical midline incision	68	63.0	All types of colorectal resections for malignant lesions of the colorectum	n/a	37	20	n/a	n/a
				Pfannensteil	24	65.8				0		
De Souza *et al.* [51]	2010	USA	Case control	Periumbilical midline incision	231	62.68	All types of colorectal resections for both benigh and malignant lesions of the colorectum	n/a	17.5	56	n/a	n/a
				Pfannensteil	139	61.32				0		
Lim *et al.* [52]	2012	Korea	Case control	Periumbilical midline incision	92	63	Left colonic resections for colorectal cancer	164.5 ± 8.6	20	2	12	n/a
				LIF transverse	55	66		167.4 ± 8.6		0	7	
Lee *et al.* [50]	2012	Canada	Case control	Periumbilical midline incision	68	63.0	All types of colorectal resections for malignant lesions of the colorectum	n/a	37	20	n/a	n/a
				LIF/RIF transverse	7	60.8				1		
Wolthuis *et al.* [53]	2011	Belgium	Case control	Periumbilical midline incision	21	35 (range 30–38)	Rectal resection for benign conditions	90 ± 5	10.3	0	1	0
				LIF transverse	21	34 (range 32–35)		105 ± 6.4	18.7	0	5	0
Gardenbroek *et al.* [54]	2012	Netherlands	Case series	Stoma site	3	21.5	Subtotal colectomy for inflammatory bowel disease	219	7	n/a	0	n/a
				Transanal	7						0	
Choi *et al.* [55]	2009	Korea	Case series	Transanal	11	53.6 ± 12.8	Robot-assisted laparosocopic anterior resection for rectal and sigmoid carcinoma	260.8 ± 62.9	1	0	1 leak	0
				Transvaginal	2			260.8 ± 62.9		0	1 bleed	0
Costantino *et al.* [56]	2012	France	Case control	Transanal	29	60.1	Left-sided colorectal resections for diverticular disease	122 ± 25.1	1	0	2	0
				Pfannensteil	23	59.5		105 ± 25.1		0	1	0

LIF = left iliac fossa, n/a = not available, RIF = right iliac fossa.

#### 2) Pfannensteil vs. periumbilical midline incision approach

**Figure 2.**
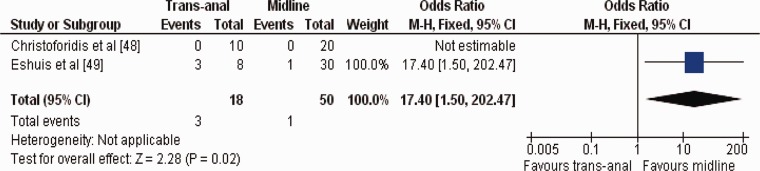
Forest plot for surgical site infection following the use of transanal *vs.* periumbilical midline incision for specimen retrieval in patients undergoing laparoscopic colorectal resections. Odds ratios are shown with 95% confidence intervals.

Two studies reported the comparison between Pfannensteil *vs.* periumbilical midline incision approach to retrieve the specimen in 462 patients undergoing LCR for both benign and malignant conditions [50, 51] (Table 4). The risk of developing incisional hernia was significantly higher (OR 53.72; 95% CI 7.48–386.04; *Z = *3.96; *P = *0.0001) following periumbilical midline incision compared to Pfannensteil incision (Figure 2).

#### 3) Periumbilical midline incision vs. transverse incision in iliac fossa

Two studies published data comparing the use of periumbilical midline incision against transverse incision in the right or left iliac fossa in 222 patients (Table 4). The risk of developing incisional hernia was significantly higher (OR 0.37; 95% CI 0.06–2.20; *Z = *1.09; *P = *0.028) following periumbilical midline incision than after transverse incision but there was no difference in the risk of SSI between the two approaches to specimen retrieval.

**Figure 3.**
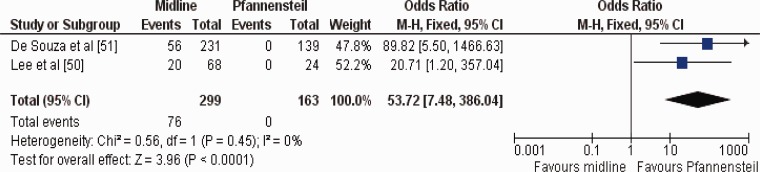
Forest plot for incisional hernia following the use of periumbilical midline *vs.* Pfannensteil incision for specimen retrieval in patients undergoing laparoscopic colorectal resections. Odds ratios are shown with 95% confidence intervals.

#### 4) Other comparisons

There was higher risk of developing SSI in the transanal approach than in the transverse incision approach [53] for specimen retrieval (Table 4). One study [54] reported specimen retrieval through the stoma site in comparison with a transanal approach, and reported no difference in SSI. One study on 13 patients reported a comparison between transvaginal and transanal approaches [55]; there were no cases of hernia or recurrence in this study. Statistically, the complication rate and duration of operation were similar in both techniques (*P = *1.0). The transanal approach was compared against Pfannensteil in a study of 52 patients undergoing left-sided laparoscopic colonic resection for diverticular disease [56]. The transanal approach was associated with slightly higher risk of SSI but operative time and incidence of incisional hernia were similar.

## SUMMARY AND CONCLUSION

Colorectal surgeons employ numerous approaches to retrieve specimens following LCR. The most common of these are periumbilical midline incision (1260 reported case in the literature), transverse incision (583 reported cases in the literature) in the right or left iliac fossa depending upon the side of colonic resection and Pfannensteil incision (293 reported cases in the literature). Periumbilical midline incision is associated with the highest risk of developing incisional hernia. There is no difference between these three common approaches to specimen retrieval, in terms of SSI. Transanal and transvaginal approaches are associated with higher risk of SSI. This conclusion is based on the combined findings of published case series, case reports and comparative studies. It may therefore be considered biased, less reliable and weaker. Randomized, controlled trials with longer follow-up are required to achieve reliable evidence before recommending the routine use of any approach for specimen retrieval in patients undergoing LCR.

**Conflict of interest:** none declared.
